# Early-Age Shrinkage Monitoring of 3D-Printed Cementitious Mixtures: Comparison of Measuring Techniques and Low-Cost Alternatives

**DOI:** 10.3390/ma19020344

**Published:** 2026-01-15

**Authors:** Karol Federowicz, Daniel Sibera, Nikola Tošić, Adam Zieliński, Pawel Sikora

**Affiliations:** 1 Faculty of Civil and Environmental Engineering, West Pomeranian University of Technology in Szczecin, 70-310 Szczecin, Poland; karol.federowicz@zut.edu.pl (K.F.); daniel.sibera@zut.edu.pl (D.S.); adam.zielinski@zut.edu.pl (A.Z.); 2 Civil and Environmental Engineering Department, Universitat Politecnica de Catalunya, 08034 Barcelona, Spain; nikola.tosic@upc.edu

**Keywords:** shrinkage measurements, 3D printed concrete, concrete curing, digital image correlation, early-age shrinkage, non-contact shrinkage measuring, strain monitoring, dimension stability, additive manufacturing in construction

## Abstract

**Highlights:**

**What are the main findings?**

- DIC, laser, LVDT, smartphone, and GoPro were evaluated for early-age shrinkage.
- Aggregate type strongly affects shrinkage and porosity.
- DIC enabled the earliest and most complete deformation monitoring.
- Laser sensors and LVDTs showed high stability in printed-element testing.
- Low-cost tools can monitor early-age shrinkage with good stability.

**What are the implications of the main findings?**

- Laser sensors offer high precision without mechanical contact with the sample.
- Low-cost cameras can substitute commercial DIC if properly stabilized and set.
- The standard linear shrinkage test is not suitable for early-age shrinkage of 3D-printed concrete.
- Results support the development of future test standards for 3D-printed concrete.

**Abstract:**

Early-age shrinkage in 3D-printed concrete constitutes a critical applied challenge due to the rapid development of deformations and the absence of conventional reinforcement systems. From a scientific standpoint, a clear knowledge gap exists in materials science concerning the reliable quantification of very small, rapidly evolving strains in fresh and early-age cementitious materials produced by additive manufacturing. This study investigates practical and low-cost alternatives to commercial optical systems for monitoring early-age shrinkage in 3D-printed concrete, a key challenge given the rapid deformation of printed elements and their typical lack of reinforcement. The work focuses on identifying both the most precise method for capturing minor, fast-developing strains and affordable tools suitable for laboratories without access to advanced equipment. Three mixtures with different aggregate types were examined to broaden the applicability of the findings and to evaluate how aggregate selection affects fresh properties, hardened performance, and shrinkage behavior. Shrinkage measurements were carried out using a commercial digital image correlation system, which served as the reference method, along with simplified optical setups based on a smartphone camera and a GoPro device. Additional measurements were performed with laser displacement sensors and Linear Variable Differential Transformer *LVDT* transducers mounted in a dedicated fixture. Results were compared with the standardized linear shrinkage test to assess precision, stability, and the influence of curing conditions. The findings show that early-age shrinkage must be monitored immediately after printing and under controlled environmental conditions. When the results obtained after 12 h of measurement were compared with the values recorded using the commercial reference system, differences of 19%, 13%, 16%, and 14% were observed for the smartphone-based method, the GoPro system, the laser sensors, and the LVDT transducers, respectively.

## 1. Introduction

### 1.1. State of the Art

Among all civil engineering processes that have been developing in recent years, one of the fields that has seen the most rapid advances is 3D-printed concrete (3DPC) [1]. This technology, often referred to as additive manufacturing, enables the creation of complex objects with cementitious mixes without traditional formwork. 3DPC utilizes a printing setup (gantry or robotic arm) controlled by computer-aided design (CAD) software. Thanks to an almost fully automated process, manual labor and construction waste are minimized [1]. Efficient use of material in hollow printed elements reduces the structure’s total weight, leading to a more sustainable and efficient approach to structural design.

3DPC enables the creation of structures that are not only aesthetically unique but also more resilient and efficient [2]. Additionally, 3D printing allows for greater design freedom, fostering architectural innovation and creativity. The applications of 3D concrete printing are diverse, ranging from residential buildings and commercial structures to infrastructure projects such as bridges and dams [3]. The technology has the potential to address housing shortages, reduce environmental impact, and accelerate construction timelines [4,5,6]. As the industry continues to explore and refine this technology, it is likely to become an integral part of the future of construction, offering sustainable and efficient solutions to the challenges faced by the built environment. However, to achieve their full potential, 3DPC structures need to be durable, an aspect tightly related to their curing conditions and microstructure.

Recent overview studies emphasize that despite rapid technological development, the lack of standardized testing methods remains one of the main barriers to the widespread adoption of 3D concrete printing. Vasilić [7] highlighted that existing standards for conventional concrete are only partially applicable to printed materials due to their anisotropy, lack of compaction, and absence of formwork. The author stressed the urgent need for dedicated testing procedures addressing early-age behavior and durability of 3DPC. Similarly, Wolfs [8] critically assessed the current maturity of 3D concrete printing and concluded that quality control and material characterization are still insufficient for reliable large-scale implementation. Particular attention was drawn to early-age material behavior, which strongly affects dimensional stability and long-term performance of printed elements.

Previous studies by Sikora et al. [9] and Mohan et al. [10] showed that 3DPC might have locally higher porosity than standard concrete. It is partly explained by the layered structure of printed elements, which tends to increase porosity in the interfacial transition zone (ITZ) and thereby decrease durability [11]. The higher porosity of 3DPC elements is strongly linked with a lack of compaction during the deposition of material (e.g., by vibration) [12]. A comprehensive review by Els et al. [13] demonstrated that shrinkage strains in 3D-printed concrete are significantly higher than those observed in cast concrete. The authors identified plastic shrinkage as the dominant early-age mechanism and pointed out that large discrepancies between reported values are largely caused by differences in measurement methods and the omission of early-age deformation. Additionally, shrinkage of 3DPC has been identified in many studies as one of the most crucial factors determining durability. 3D-printed concrete exhibits plastic, drying, and autogenous shrinkage, each driven by distinct moisture-related mechanisms. Plastic shrinkage appears soon after deposition due to rapid surface water loss, while drying shrinkage develops later as water evaporates from the hardened matrix [14]. Autogenous shrinkage occurs without external moisture loss and results from self-desiccation during hydration, especially in low water–binder mixes [15]. Experimental investigations by Han et al. [16] further showed that plastic shrinkage in 3DPC is strongly influenced by the self-weight of upper layers and internal restraint conditions. Their results confirmed that early-age shrinkage strains can reach very high values and significantly increase the risk of cracking, particularly in multilayer printed elements. Nonetheless, only a few studies tackled the challenge of measuring this phenomenon. Such a significant lack of fundamental knowledge of how shrinkage develops in 3DPC may stem from the absence of a standardized procedure or an accurate, practical method for determining deformation in the plastic state. For 3DPC, plastic deformations in the first hours are critical because the material is exposed and has no formwork to limit early evaporation. It increases the risk of rapid moisture loss and early-age instability.

Bekaert et al. [17] tested total shrinkage on printed 40 × 60 × 500 mm samples, with deformation recorded after the first 24 h, resulting in almost 1100 μm/m after 28 days. Since the measurements started after 24 h, drying in the plastic state was not taken into account. Le et al. [18] tested 3D printable concrete using a standard method complying with EN 12617-4:2002 [19] on cast 75 × 75 × 229 mm specimens. Total shrinkage after 30 days was 597 μm/m. In this method, the influence of the printing process on shrinkage is not considered, and plastic shrinkage during the first 24 h is completely neglected. Zhang et al. [20] also tested shrinkage on printed specimens, but only after the first 24 h of initial hardening, resulting in a total deformation of 826 μm/m after 210 days. The specimens were stored in a climatic chamber, and the measurements again started after material hardening; therefore, changes in the plastic phase were not considered. A different approach was presented by Moelich et al. [21], who used digital image correlation to measure the plastic deformation of freshly printed specimens. Tests were conducted on 300 mm-long printed samples for the first 4 h, and the resulting deformation reached 10,000 μm/m. Those results are in line with previously published research [22], which showed that printed samples also suffered plastic deformation above 6000 μm/m after 2 h of hardening. It is significantly higher than the deformation measured on standard specimens or in research where measurements began after the first 24 h. However, the method had several limitations. Measurements were performed only on the top layer of a multilayer specimen. The sample was stabilized using a rod embedded at mid-length to increase stability. In addition, the specimen was placed in a climatic chamber at 23 °C, which is slightly higher than the 20 °C temperature commonly assumed in standards. Markin and Mechtcherine [23] systematically reviewed and compared experimental methods for measuring plastic shrinkage and plastic shrinkage cracking in 3D-printed concrete. They demonstrated that contactless optical techniques, particularly DIC-based methods, are well suited for capturing early-age deformations, which are otherwise inaccessible using conventional measurement approaches.

Mechtcherine et al. [24] presented a roadmap for testing 3DPC. The authors suggested that physical, mechanical, and durability aspects should be tested. The importance of determining shrinkage was strongly addressed, but no clear practical recommendations were provided. It clearly shows that techniques for measuring 3DPC shrinkage need to be critically evaluated, tested, and compared; hence, this is the main objective of this study. From a regulatory perspective, Kreiger et al. [25] emphasized that the lack of reliable and comparable experimental data remains a key obstacle in developing acceptance criteria for additively manufactured concrete structures. The authors highlighted the need for robust, reproducible testing methods that reflect real printing conditions, including early-age deformation and durability-related phenomena. Although studies on the shrinkage of 3DPC are available in the literature, most authors rely on standard testing methods. Measurements are usually performed after 24 h, and the specimens are kept in molds that differ significantly from the conditions experienced by printed structures. Therefore, it is necessary to apply methods that enable strain measurements during the first 24 h.

### 1.2. Significance and Novelty

The novelty of this study lies in assessing low-cost alternatives to commercial optical measurement systems, which typically require complex, expensive equipment. Reliable monitoring of early-age shrinkage in 3D-printed cementitious materials remains a scientific and practical challenge, as deformations are small, occur rapidly, and strongly depend on mixture composition and early curing conditions. Existing standardized methods are often not well suited to capture these effects in additively manufactured elements, especially during the fresh and early hardening stages. Testing several measurement devices was important for two reasons. First, it allowed identification of the most precise and stable method for monitoring early-age shrinkage in 3D-printed concrete, where deformations are small and occur rapidly. Second, it enabled the evaluation of simple, inexpensive tools suitable for laboratories with limited access to advanced equipment. To broaden the relevance of the results, three mixtures with different aggregate compositions were examined: a conventional 3D-printable mix and two variants with modified aggregates to study their influence on fresh behavior, hardened properties, and shrinkage performance.

The scientific problem addressed in this study is the lack of validated, accessible measurement techniques capable of reliably capturing early-age shrinkage in 3D-printed cementitious mixtures. The main goal of the research is to compare the accuracy, stability, and practical applicability of selected contact and non-contact measurement methods, with particular emphasis on low-cost optical solutions. The specific objectives are: to benchmark simplified optical techniques against a commercial digital image correlation system, to quantify differences between optical, laser-based, and contact measurements, to evaluate the suitability of these methods for early-age monitoring of 3D-printed elements, and to analyze the effect of aggregate modification on shrinkage behavior. A commercial DIC system (Aramis 3D Camera by Zeiss, Oberkochen, Germany) served as the reference method. Alongside it, two simplified optical approaches using a smartphone camera (Sony Xperia XA1, Sony Corporation, Tokyo, Japan) and a GoPro HERO 8 (GoPro, Inc., San Mateo, CA, USA) camera were tested. Laser sensors (Omron ZX1, Omron Ayabe Factory, Ayabe, Japan) and LVDT transducers (HBM WA100, Hottinger Baldwin Messtechnik GmbH (HBM), Rosenfeld, Germany) in a custom setup provided additional non-contact and contact measurements. The obtained results were compared with the standard linear shrinkage test according to EN 12390-16 [26], thereby verifying the precision, stability, and practical usability of each method and demonstrating the potential of low-cost solutions as viable alternatives.

## 2. Materials and Printing Setup

### 2.1. Materials

**Figure 1:**
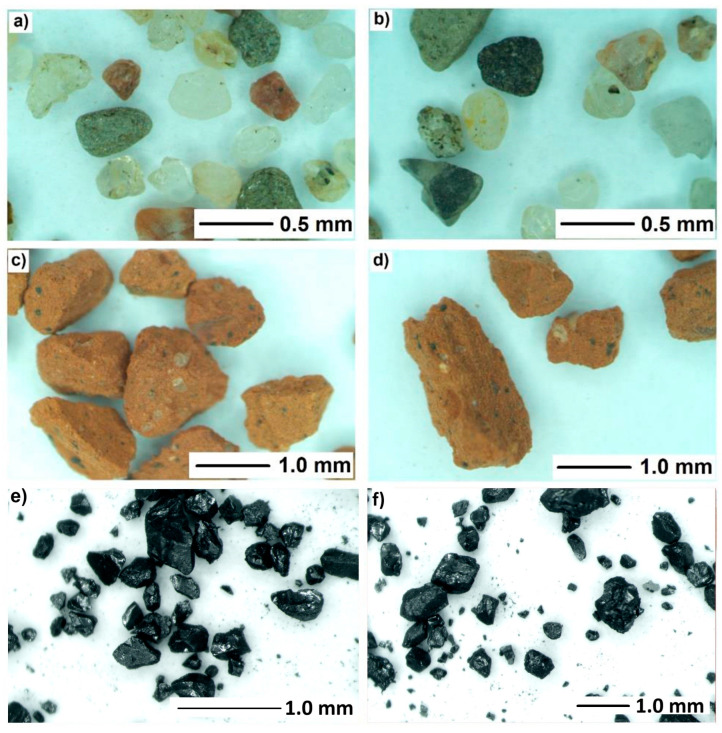
Optical microscope images of river sand (**a**,**b**), RMA (**c**,**d**), and magnetite sand (**e**,**f**), reproduced from [27].

The 3DPC mixtures used in this study were previously developed and published by the authors [27,28]. The mixture designs presented in Table 1 were used, with slight adjustments to the composition, required due to the new raw materials introduced in this study compared with those in previous studies. All three mixes had the same binder composition: cement CEM I 42.5R (Heidelberg Group, Heidelberg, Poland), fly ash class F (ImmerBau Sp. Z o.o., Poznań, Poland), and silica fume (Mikrosilika Trade Company, Poznań, Poland). Aggregates used in the test had a maximum particle size of 2 mm and consisted of river sand (SKSM, Szczecin, Poland), magnetite sand (LKAB, Kiruna, Sweden), and recycled masonry aggregate (CEGMAR, Cegłów, Poland). The aggregates are shown in Figure 1, reproduced from [27]. The replacement rate for natural aggregate was set to 20 vol%. Because of the high porosity of the recycled masonry aggregate (RMA), extra water was added to compensate for its water absorption. For RMA, 24 h water absorption was 12.7% [27], and in research, 60% of this value was added as extra water. The magnetite aggregate exhibited water absorption comparable to that of natural aggregate, so no additional water was required. Its more angular shape, however, required an adjustment of the superplasticizer dosage. An effective water-to-binder ratio of w/b = 0.24 was used for all mixes. To achieve proper workability and printability parameters, a polycarboxylate superplasticizer, Sika ViscoCrete 111 (Sika, Baar, Switzerland), was used.

### 2.2. Mixing and Printing

**Figure 2:**
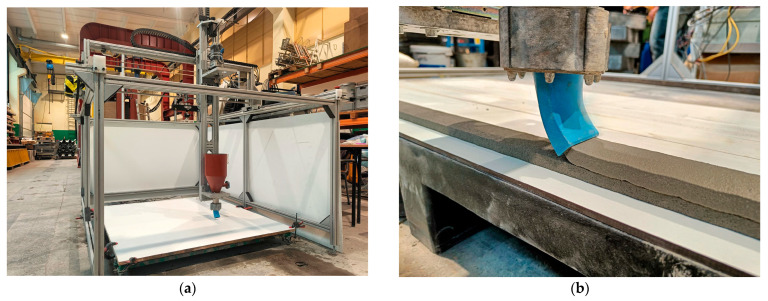
Printing setup: (**a**) 3D printer; (**b**) sample during printing process.

The mixtures were prepared in a standard laboratory 100 L concrete mixer with a rotation speed of 29 rpm. The mixing procedure was divided into two stages. First, all dry components were mixed for at least 120 s until homogeneous. After that, water was added, and mixing continued for up to 10 min. Subsequently, the prepared material was manually transported to an extrusion 3D printer. A self-build Cartesian 3D printer was used for printing, with parameters described in detail in previous publications [28]. It has a printing space of about 140 × 140 × 80 cm. The concrete mix is delivered by a Swing M pump to a rectangular nozzle with a 40 × 10 mm cross-section. The system uses G-code as its programming language. Shrinkage test specimens were printed as multilayer elements composed of six layers, each 10 mm high, with a total specimen length of 500 mm. The adopted geometry enabled measurements that accounted for rheological effects, such as plastic creep, and verified the buildability parameter of the mixtures. The 3D printer used and the specimens during the printing process are shown in Figure 2.

## 3. Testing Procedures

**Figure 3:**
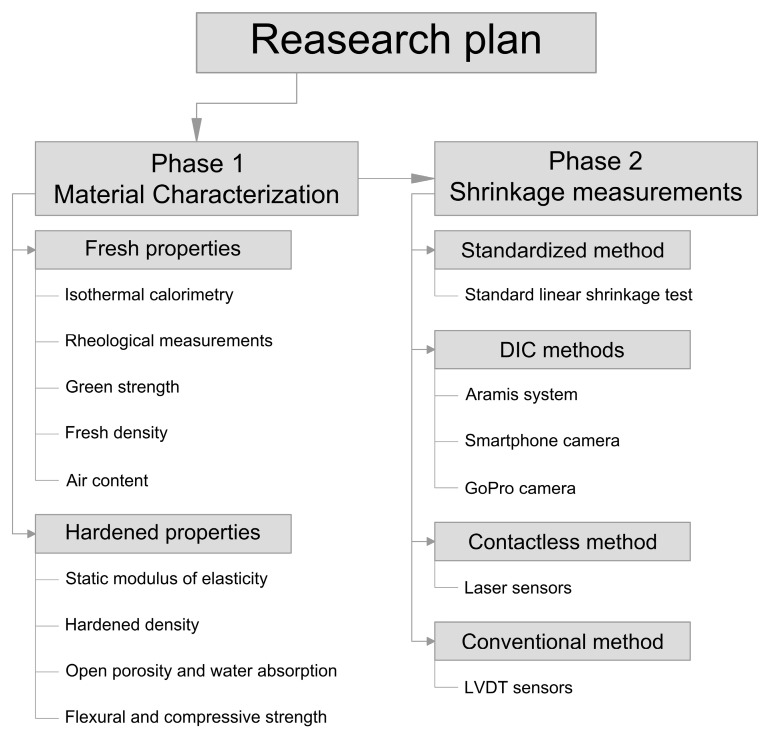
Graphical scheme of the research plan.

To ensure a clear structure, the methodology was divided into two phases. Phase 1 covers material characterization, including tests of fresh and hardened properties. Phase 2 focuses on shrinkage behavior and the evaluation of different measurement techniques for early-age deformation monitoring. To better illustrate the workflow, a graphical scheme of the research plan is presented in Figure 3.

### 3.1. Phase 1—Material Characterization

#### 3.1.1. Isothermal Calorimetry

The calorimetric test was carried out using an TAM Air 8-channel isothermal calorimeter (TA Instruments, New Castle, DE, USA) to monitor the heat evolution of the cementitious mixtures. To ensure repeatability and accuracy of the measurements, 1 L batches of the mix were prepared for calorimetric testing. The mixing procedure was similar to that described in Section 2.2, except that a standard planetary mortar mixer was used instead of a 100 L concrete mixer. The mixing time and the sequence of component addition remained unchanged. Approximately 100 g of fresh mix was then placed in sealed test containers to prevent moisture exchange with the environment. The samples were immediately inserted into the calorimeter, which maintained a constant temperature throughout the measurement period. The heat flow rate was continuously recorded over time to capture the hydration kinetics of the cementitious system. The obtained results were normalized to the mass of the tested sample.

#### 3.1.2. Rheological Measurements

To assess the influence of aggregate type on printability and to better understand potential differences in mixture behavior during printing, a series of rheological tests was performed. Rheological tests were conducted using a measuring cell with a volume of 120 mL. Three types of tests were selected: determination of the static yield stress (SYS, related to mixture stability after printing), thixotropy (affecting pumpability), and recovery rate (stability of parameters before and after pumping).

The static yield stress (SYS) was measured at 5 min intervals, starting 15 min after the water-cement contact. Each measurement was preceded by a pre-shear at 100 s^−1^ to achieve a homogeneous structure. The actual measurement was performed over 60 s at a constant shear rate of 0.1 s^−1^. Thixotropy was evaluated using a hysteresis loop test. As with SYS, the measurement was preceded by pre-shearing. The shear rate was ramped from 0.1 s^−1^ to 100 s^−1^ and then returned to 0.1 s^−1^ to determine the loop area and thixotropic behavior. The recovery rate was assessed by determining SYS before and after intensive shearing simulating pumping. In the first stage, the sample was sheared for 60 s at 0.1 s^−1^, then at 100 s^−1^ for 30 s, and finally again at 0.1 s^−1^ for 60 s. The recovery rate was calculated as the ratio of SYS after shearing to the initial SYS and expressed as a percentage.

#### 3.1.3. Green Strength

As a preliminary test to qualify mixtures for printability, green strength measurements were conducted. Green strength was evaluated on cylindrical specimens with dimensions of 120 × 60 mm. An electromechanical testing machine with a maximum force capacity of 500 N and precise displacement measurement was used. Force and displacement data were recorded using a QuantumX amplifier (Hottinger Brüel & Kjær, Darmstadt, Germany) at a frequency of 5 Hz. During testing, the crosshead was displaced at a constant rate of 0.25 mm/s. The specimens were cylindrical, measuring 60 mm in diameter and 120 mm in height. Tests were performed at 30, 45, 60, and 75 min after mixing to evaluate the early development of material strength.

#### 3.1.4. Fresh Density, Air Content

The density of the fresh concrete mix was measured using a 1 L graduated cylinder in accordance with EN 1015-6:2000 [29]. The cylinder was filled in layers, with each layer properly compacted. Measurements were carried out twice: first for the mix directly after mixing, and then after it passed through the pump. Additionally, the air content of the fresh mix was determined in accordance with EN 1015-7:1998 [30], both before and after the printing process.

#### 3.1.5. Static Modulus of Elasticity

The static modulus of elasticity of concrete was determined on cylindrical specimens with dimensions of 100 × 200 mm. The specimens were tested at 1, 3, and 28 days of age to evaluate the early and later-age stiffness development.

The test was conducted using mechanical dial gauges mounted longitudinally along the cylinder’s height to measure axial deformations. The specimens were placed in a universal testing machine and subjected to uniaxial compressive loading. A preloading step of approximately 10% of the estimated ultimate load was applied to ensure full seating of the specimen and measurement system. The load was then increased gradually at a constant rate, and the corresponding axial strains were recorded from the dial gauges. The modulus of elasticity was calculated according to the standard procedures outlined in EN 12390-13 [31].

#### 3.1.6. Hardened Density, Open Porosity, and Water Absorption

The hardened density of the concrete was determined using geometrical measurements of the specimens prepared for mechanical testing. Each specimen was measured in three perpendicular directions using a caliper with an accuracy of 0.1 mm. The volume was calculated from the measured dimensions, and the specimen’s mass was recorded. Hardened density was calculated as the mass divided by the calculated volume.

Open porosity and water absorption were determined according to a standard saturation procedure. The specimens were first dried in an oven at 105 ± 5 °C until a constant mass was achieved and then cooled to room temperature in a desiccator. The dry mass was recorded. Afterward, the specimens were immersed in water for 48 h to ensure complete saturation. Excess surface water was removed with a damp cloth, and the saturated mass was measured. Finally, the specimens were weighed while suspended in water to obtain the hydrostatic mass.

#### 3.1.7. Flexural and Compressive Strength

Flexural and compressive strength tests were performed on cast mortar specimens with dimensions of 40 × 40 × 160 mm. The 3D-printable material was first pumped through the printing system, after which samples were also prepared using conventional casting methods. Specimens were cured in molds for 24 h at 20 °C, then demolded and stored in a humid chamber at 20 °C until testing.

Flexural strength was determined according to EN 1015-11 [32] using a three-point bending setup with a loading rate of 50 N/s. For each testing day, six samples were prepared. After the flexural test, the two resulting halves were used to measure compressive strength according to the modified procedure in EN 1015-11 [32], with a loading rate of 0.6 MPa/s. Tests were carried out at standard ages of 1, 3, 7, and 28 days.

### 3.2. Shrinkage Measurement

Shrinkage behavior is a critical aspect in 3D-printed concrete, as printed elements often experience significant early-age deformations and typically contain no reinforcement. In simplified terms, shrinkage can be divided into plastic, autogenous, and drying shrinkage, each governed by different mechanisms and characterized by distinct development rates. In printed structures, many studies focus on only one type of shrinkage due to limitations of conventional measurement methods. However, when considering the actual service conditions of printed elements, it is necessary to account for total shrinkage, defined as the combined deformation that occurs from the end of printing until the application of external loading.

In this study, digital image correlation (DIC) was selected as the reference method, as it is the most frequently reported optical approach in the literature beyond standardized tests. DIC enables measurements to begin in the plastic state without disturbing the specimen geometry, though its accuracy depends strongly on camera resolution and lighting conditions. As an alternative to DIC, laser displacement sensors and conventional LVDT transducers were also investigated. The LVDT measurements were made possible by a custom-designed plastic holder embedded directly into the specimen during printing. No external curing was applied to better reproduce the environmental exposure typical of on-site printed elements.

#### 3.2.1. Standard Linear Shrinkage Test

**Figure 4:**
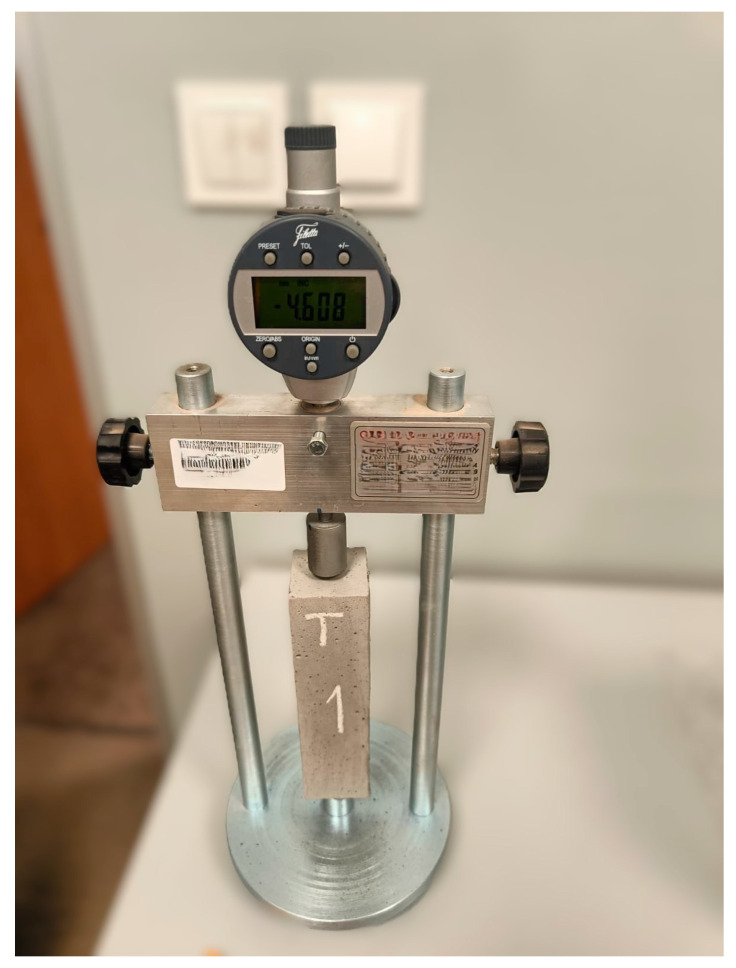
Set up for the standard linear shrinkage test.

The Linear Shrinkage Test (LST) method involves preparing standard mortar specimens in molds measuring 40 × 40 × 160 mm and is based on EN 12390-16 [26]. Steel reference points are embedded at both ends of each specimen. After 24 h of curing in molds, the specimens are placed vertically in a measuring frame equipped with a dial gauge. An example of the testing setup and a specimen with embedded reference points is shown in Figure 4.

The dial gauge records the change in specimen length, from which the total shrinkage is calculated using the following formula:

(1) $$\varepsilon_{tot}=\frac{\Delta L}{L}$$

where

- $\varepsilon_{tot}$—total shrinkage (µm/m).

- ΔL—measured length change (µm).

- L—initial specimen length (constant value of 160 mm).

Measurements were taken after 1, 2, 3, 7, 14, 21, and 28 days. For each mixture, three specimens were tested, and the mean value of shrinkage was calculated.

#### 3.2.2. Digital Image Correlation with Commercial Setup

**Figure 5:**
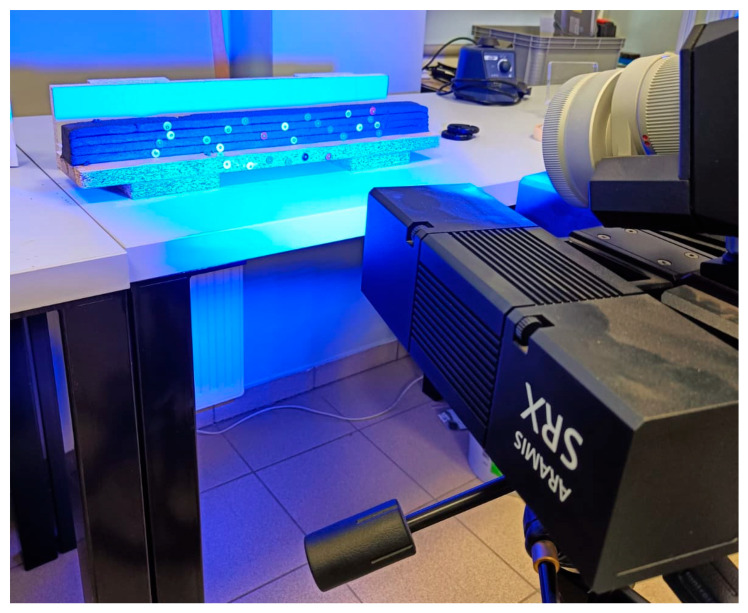
Set up for the Aramis system.

An Aramis SRX system (GOM GmbH, a ZEISS company, Braunschweig, Germany) was employed in this study. In the Aramis system, surface deformation is analyzed using digital image correlation (DIC), which relies on a distinctive surface pattern. Depending on the specimen characteristics and the required measurement resolution, the system can operate using either applied speckle patterns or dot markers. A specimen during measurement is shown in Figure 5.

The system used two synchronized 4K-resolution cameras, each mounted on a rigid measuring beam with an inter-camera spacing of 180 mm. The cameras were positioned approximately 400 mm from the test specimens, providing optimal focus and measurement accuracy. This configuration defined a measurement volume of 290 mm × 230 mm × 200 mm (width × height × depth).

For the tested samples, adhesive point markers were applied to the specimen surfaces to enable precise tracking of displacements at key control points. The displacements of each marker were recorded and analyzed to assess the local deformation behavior and rigidity of the samples. The combination of high-resolution 4K imaging, accurate calibration, and careful marker placement ensured high spatial accuracy and measurement repeatability.

#### 3.2.3. Digital Image Correlation with Alternative Camera Systems

As an alternative to the Aramis system, strain measurements were also obtained using a standard smartphone camera and a GoPro sports camera. The selection of these devices was intentional, as they employ different lens configurations—with the GoPro using a wide-angle lens. Both cameras operated in continuous mode, capturing images at 5 min intervals. In both cases, automatic settings for focus, white balance, and exposure were used. This configuration was deliberately selected to represent a realistic, low-effort laboratory scenario using readily available consumer devices with default settings, rather than an optimized photogrammetric setup. The selected acquisition interval of 5 min was based on previous studies [14,15,21,22] on early-age deformation of 3D-printed concrete and on the expected rate of shrinkage development within the first 12–24 h, allowing the main deformation trends to be captured while limiting data redundancy and storage demands.

**Figure 6:**
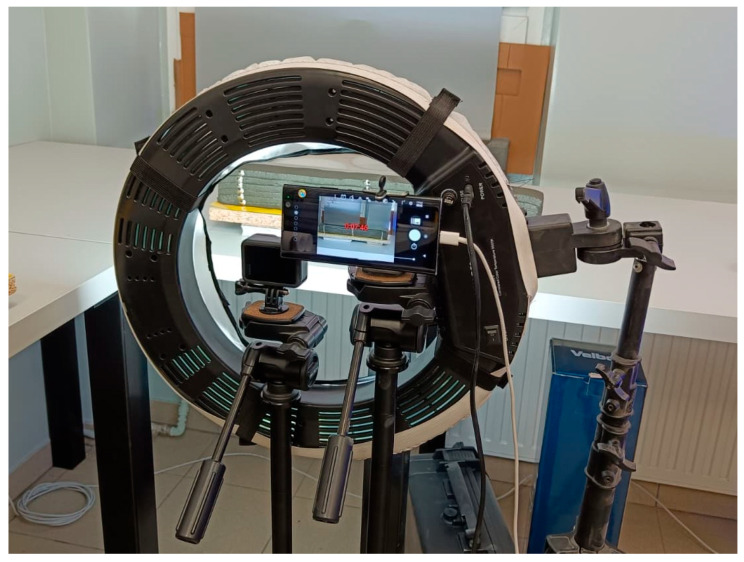
Set up for an alternative DIC system.

To ensure proper illumination, an external photographic lamp was used, and the entire experimental setup is shown in Figure 6. Similarly to the Aramis system, fixed reference points were applied. Because there was no automatic sample size detection function, a reference scale with known dimensions was placed on the platform to enable later image scaling.

Analogous to the measurements performed with the Aramis system, samples 500 mm long were used, consisting of six layers with a cross-section of approximately 40 × 10 mm.

#### 3.2.4. Laser Sensor Measurement

**Figure 7:**
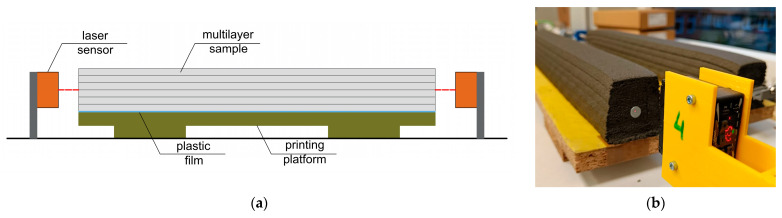
Laser sensors measuring setup: (**a**) schematic graph; (**b**) sample during measurement.

Another variant of the non-contact measurement method uses laser sensors. In this case, it was not possible to measure surface shrinkage, but only to determine the average linear shrinkage of the entire specimen. The setup was based on the test stand described in detail by Federowicz et al. [33]. OMRON ZX1-LD50 (Omron Corporation, Kyoto, Japan) sensors were used, offering a measurement accuracy of 2 µm and a ±10 mm measurement range. The sensors were connected to a Universal Measuring Amplifier QuantumX MX840B (Hottinger Brüel & Kjær, Darmstadt, Germany). Measurements were recorded at 60 s intervals (due to software limitations). A schematic of the experimental setup and the specimen during measurement is shown in Figure 7.

In order to ensure direct reference to the results reported in the cited study, the specimen was placed in a climatic chamber with controlled relative humidity (RH = 70%). This condition differs from the remaining specimens investigated in this study, which were stored under ambient laboratory conditions (RH ≈ 30%). In addition, a different specimen length of 1350 mm was adopted, allowing both a direct comparison with the literature data and an assessment of the influence of specimen length on the recorded shrinkage values. Due to these deliberate methodological differences, only the evolution of shrinkage trends and the overall material behavior can be meaningfully compared, providing valuable qualitative insight into the shrinkage development of 3D-printed concrete.

#### 3.2.5. LVDT-Based Shrinkage Measurement

**Figure 8:**
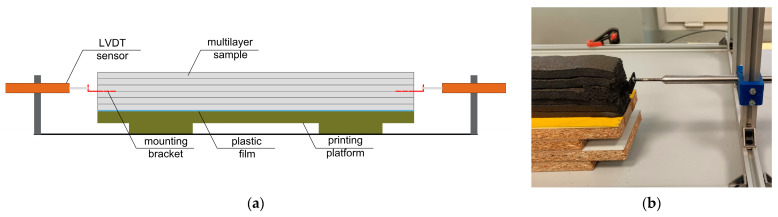
LVDT sensors measuring setup: (**a**) schematic graph; (**b**) sample during measurement.

The final measurement setup used in the presented research was based on LVDT sensors with a 100 mm gauge length manufactured by Hottinger Baldwin Messtechnik GmbH (Darmstadt, Germany). To enable connection between the sensor and the specimen, a custom L-shaped mounting bracket with an embedded length of 30 mm was designed and inserted between the third and fourth printed layers during the printing process. The mounting bracket included additional protrusions to improve adhesion to the concrete, and its geometry was optimized to avoid interference with the specimen’s shrinkage deformation. A schematic of the setup and the specimen during testing is shown in Figure 8. As with the laser sensor measurements, linear deformation was recorded and used to determine the average total shrinkage. The specimen geometry remained constant at 500 × 40 × 60 mm.

## 4. Results

### 4.1. Phase 1—Material Characterization

#### 4.1.1. Isothermal Calorimetry

**Figure 9:**
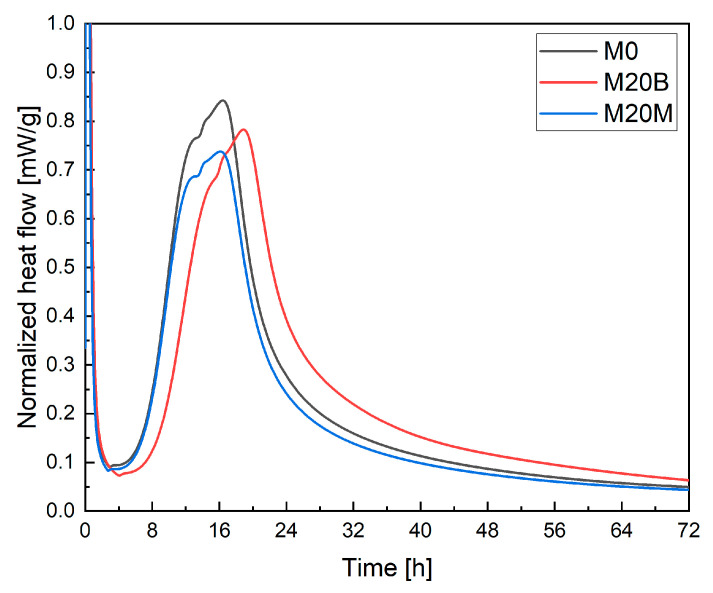
Isothermal calorimetry results.

The calorimetric results (Figure 9) indicate noticeable differences in the hydration kinetics between the analyzed mixtures. The reference sample M0 exhibited the most prominent exothermic peak, occurring at approximately 15–17 h, corresponding to the main acceleration phase of hydration. In contrast, the M20B mixture showed a lower peak intensity and a delay in its onset of around 2–3 h. This behavior suggests a partial retardation of early hydration reactions. The delayed occurrence of the central exothermic peak in M20B can be attributed to the presence of porous aggregate, which absorbs additional mixing water and gradually releases it during later stages of hydration. This process provides internal curing, maintains internal moisture, and supports continuous hydration. However, in the early phase, reduced free water availability limits ion mobility and slows the dissolution of clinker minerals, thereby extending the induction period and delaying the onset of the acceleration stage. As hydration progresses, the slow release of water from the pores reactivates the reactions, resulting in a prolonged and broader heat flow curve. Consequently, M20B shows slower but more sustained heat evolution than M0. The M20M mixture, in turn, exhibited the lowest peak magnitude with almost no time delay, indicating that partial incorporation of magnetite sand reduces reaction intensity without significantly altering the hydration kinetics. Overall, the results suggest that the internal curing effect in M20B delays the early hydration process while enhancing long-term reaction stability, as reflected in the extended heat-release profile.

#### 4.1.2. Rheological Measurements

**Figure 10:**
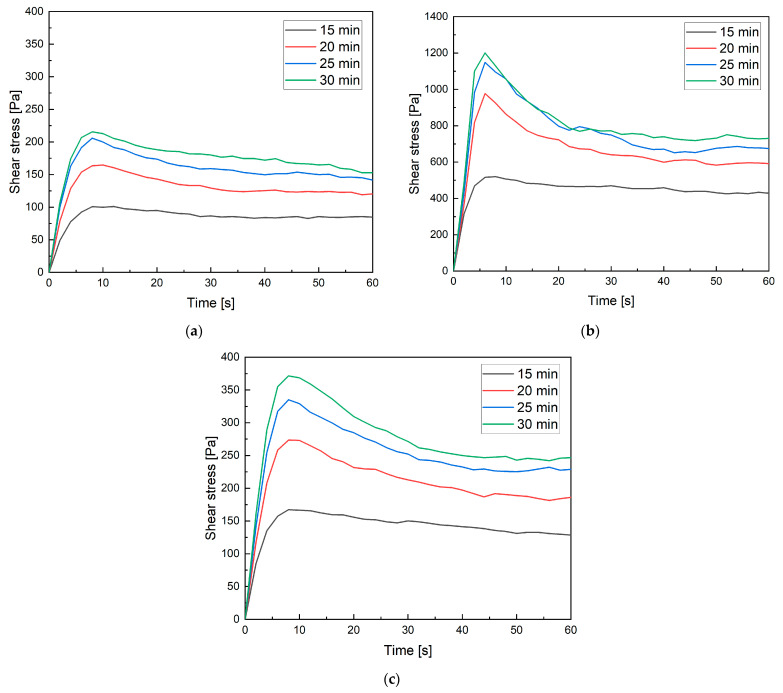
Static yield stress results: (**a**) M0; (**b**) M20B; (**c**) M20M.

The results of the static yield stress (SYS) tests are presented in Figure 10a–c. The parameter’s development during the first 15 min after mixing is shown. In this period, the first reactions occurred in the mixtures, enabling the formation of rheological properties necessary for 3D printing. Mixtures M0 and M20M exhibited similar parameters during the initial measurement stage. Later, the differences increased, highlighting the influence of aggregate shape on the rheological behavior of the mixtures. The M20M mixture, containing crushed aggregate with sharp edges, demonstrated greater shape stability compared to the mixture with pure sand (M0). In the case of mixture M20B, a clear difference was observed: the SYS was higher from the very beginning compared to the reference mixture. This effect is associated with the absorption of free mixing water by the porous ceramic aggregate. In addition, the shape of the ceramic aggregate grains further intensified the differences in rheological behavior.

**Figure 11:**
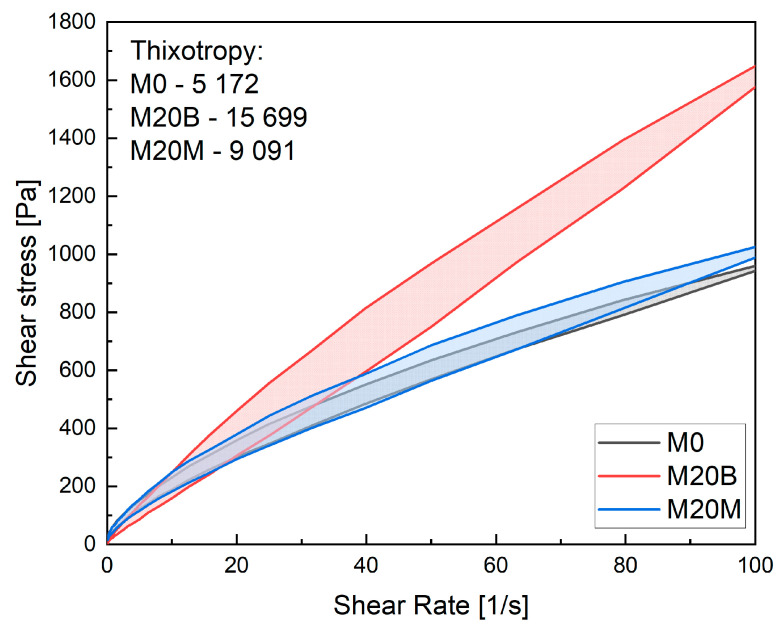
Hysteresis loop test results.

The thixotropy results (Figure 11) indicate significant differences in structural rebuilding behavior among the tested mixtures. The reference mixture M0 exhibits the lowest thixotropic area (5172 Pa·s), reflecting limited structural recovery during rest. The mixture M20B, where sand was partially replaced with porous recycled aggregate and additional water was added, shows the highest thixotropy value (15,699 Pa·s). It suggests substantial structural rebuilding, likely caused by higher water absorption and internal curing effects that enhance particle flocculation and cohesion between binder phases. In contrast, M20M, with partial replacement of sand by magnetite aggregate, presents an intermediate thixotropy (9091 Pa·s). The angular and dense magnetite particles likely improved mechanical interlocking but reduced the surface area available for water absorption, limiting thixotropic development compared to M20B.

Confirmation of these results can also be seen in the recovery rate. For the mixtures M0 and M20M, results were similar, reaching 63% and 62%, respectively. In the case of mixture M20B, the recovery rate reached 110%, which is associated with the presence of porous aggregate. During shearing, this aggregate absorbed additional mixing water, thereby increasing shear stresses. A recovery rate above 100% may also be interpreted as structural build-up rather than simple recovery.

#### 4.1.3. Green Strength

**Figure 12:**
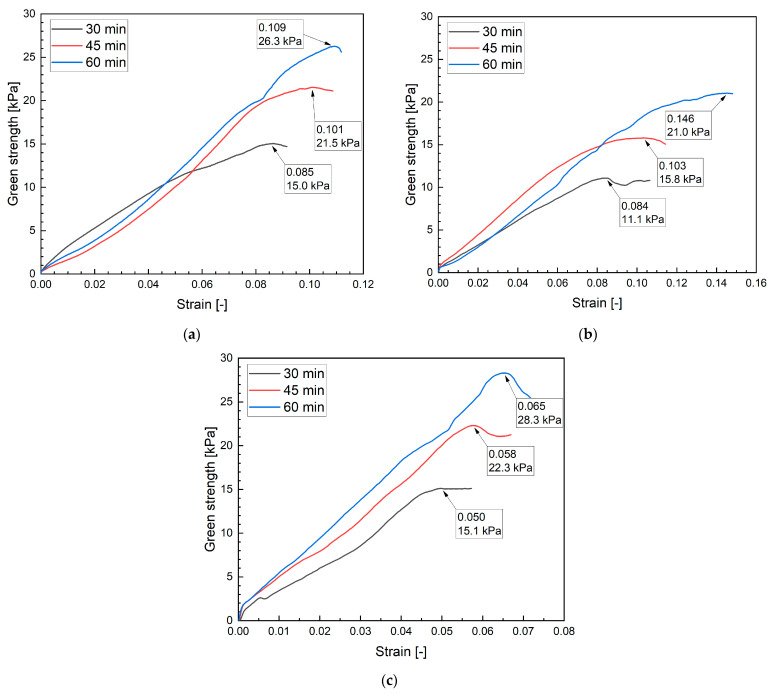
Green strength development of: (**a**) M0; (**b**) M20B; (**c**) M20M.

The development of green strength was investigated at 15 min intervals. To maintain constant measurement intervals, the first test was performed 30 min after water–cement contact. At this time, all mixtures were sufficiently stable to allow testing. For the reference mixture, the results are presented in Figure 12a–c. In subsequent intervals, a continuous increase in strength is observed, reflecting ongoing hydration reactions in the mixture. The failure stresses range from 15 kPa to 26.3 kPa, with relatively similar strains of 0.085–0.109. The reference mixture did not exhibit noticeable initial settlement, and the stress–strain curves are predominantly linear, indicating a plastic-like behavior.

The results for mixture M20B (Figure 12b) show a steady increase in green strength, with failure stresses of 11.1 kPa at a strain of 0.084, 15.8 kPa at 0.103, and 21.0 kPa at 0.146. Compared to the reference mixture, M20B reaches lower ultimate stresses but sustains significantly higher strains at failure, indicating a more ductile response. The curves are less linear, especially at longer times, reflecting greater plastic deformation capacity. This behavior is associated with the porous ceramic aggregate, which absorbs mixing water and promotes internal curing, enhancing deformability but reducing stiffness and ultimate strength.

For mixture M20M (Figure 12c), the results indicate a progressive increase in green strength, with failure stresses of 15.1 kPa at a strain of 0.050, 22.3 kPa at 0.058, and 28.3 kPa at 0.065. Compared to both the reference mixture and M20B, M20M exhibits the highest stresses at relatively low strains, indicating a stiffer, more brittle response. The curves remain close to linear over most of the loading path, followed by a clear peak and drop in strength, especially at longer times, reflecting limited plastic deformation capacity. This behavior is attributed to the angular and dense magnetite aggregate, which enhances mechanical interlocking and stiffness, resulting in higher load-bearing capacity but reduced ductility compared to mixtures with natural or porous aggregates.

#### 4.1.4. Fresh and Hardened Density and Air Content

**Figure 13:**
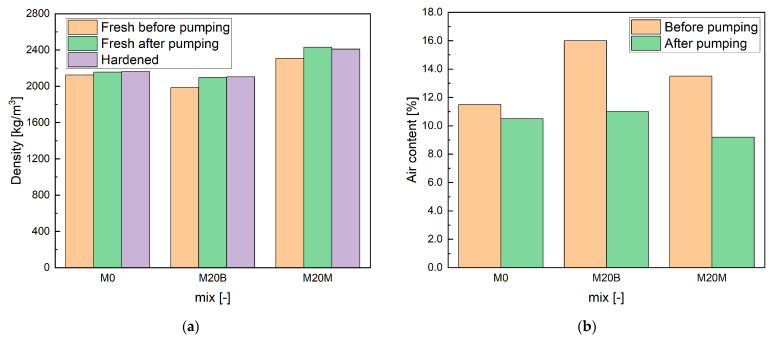
Fresh-state material properties: (**a**) mixture density before and after pumping, as well as hardened density; (**b**) air content.

The measurement of the fresh mixture density revealed minor differences, attributed to the pumping process. The results are presented in Figure 13a. Due to the use of a rotor–stator pump, which allows a maximum aggregate size of 3 mm, the mixture was subjected to additional compression as it passed through the pump. This effect was observed across all mixtures, with increases in fresh density ranging from 1.5% to 5.6%.

The fresh density ranged from 2150 kg/m^3^ for mixture M0 to 2100 kg/m^3^ for M20B, which reflects the higher porosity of the recycled aggregate, and increased to 2400 kg/m^3^ for M20M due to the use of magnetite. The density of the hardened material remained close to the fresh values. Typically, plastic-state samples have densities roughly 10% higher than those of hardened specimens. In this study, however, the hardened samples were stored in water and absorbed moisture, increasing their mass and reducing the expected difference between the fresh and hardened densities.

The results of air content in the fresh mixtures are presented in Figure 13b. A similar trend to that observed for the mixture densities can be noted. Mixture M20B, which contains a higher proportion of recycled aggregate with greater porosity, exhibited the highest overall porosity of the fresh mix. The pumping process also reduced the initial air content, which ranged from 9.2% to 11.5% across all mixtures.

#### 4.1.5. Static Modulus of Elasticity

**Figure 14:**
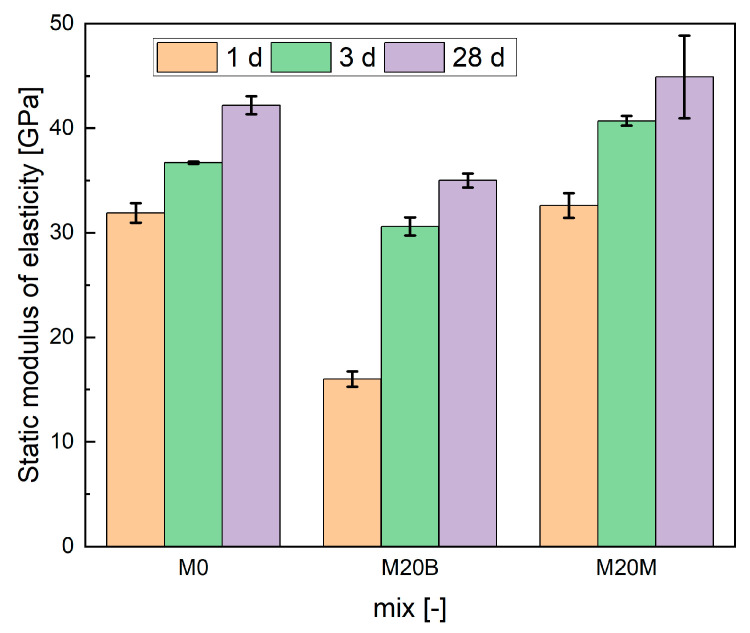
Development of the static modulus of elasticity.

Figure 14 presents the development of the elastic modulus over time. Mixtures M0 and M20M show comparable values after the first 24 h, with a slight advantage for M20M that can be attributed to the presence of crushed aggregate. The significant difference between these mixtures and M20B results from the porous structure of the ceramic aggregate and its lower strength, both of which inherently reduce material stiffness. At later testing ages, this difference becomes relatively minor, which can be explained by the water-transfer effect associated with moisture release from the ceramic aggregate.

#### 4.1.6. Open Porosity and Water Absorption

**Figure 15:**
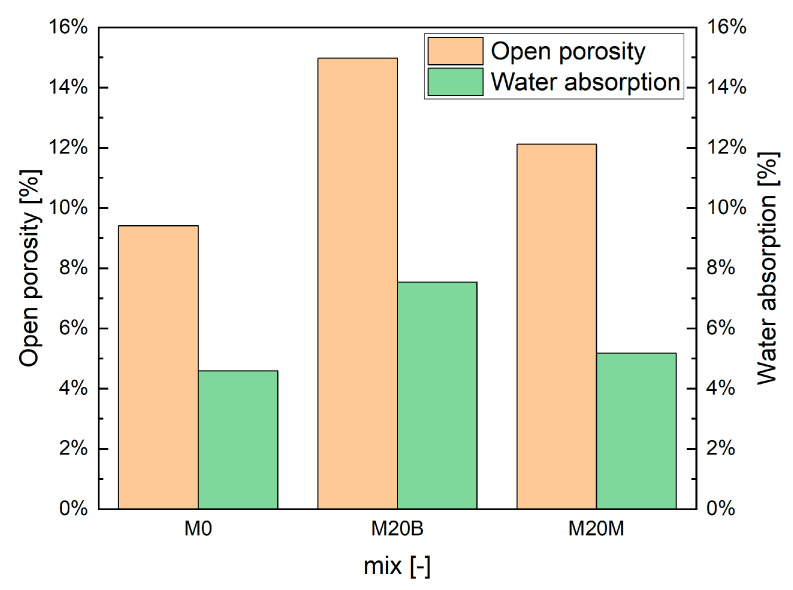
Open porosity and water absorption results.

The relationships observed in the air content of the mixtures are also reflected after printing. The measured open porosity and water absorption (Figure 15) clearly show the effect of the printing process, with the absence of proper compaction increasing the total pore volume and, consequently, the material’s water-absorption capacity. As in the air-content results, the highest open porosity was recorded for mixture M20B, which contains porous recycled aggregate. In contrast, mixtures with dense aggregates such as quartz sand or magnetite exhibit significantly lower porosity and water absorption.

#### 4.1.7. Flexural and Compressive Strength

**Figure 16:**
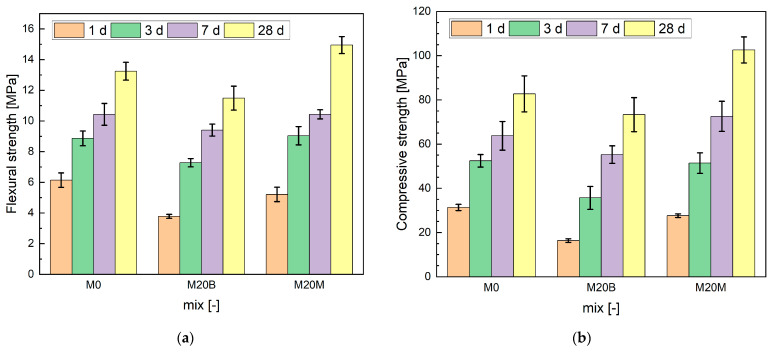
Results of mechanical tests: (**a**) flexural strength; (**b**) compressive strength.

The flexural and compressive strength results show apparent differences among the mixtures and are presented in Figure 16a,b. At an early age, M0 demonstrated the highest flexural strength, while M20B was markedly weaker; however, over time, M20M developed superior values, reaching 15.0 MPa at 28 days compared to 13.2 MPa for M0 and 11.5 MPa for M20B. A similar trend was observed in compression: after 1 day, M0 (31.3 MPa) and M20M (27.6 MPa) were significantly stronger than M20B (16.3 MPa), but at later ages, M20M surpassed all mixtures, achieving 102.6 MPa at 28 days against 82.7 MPa for M0 and 73.3 MPa for M20B. These results indicate that the use of magnetite aggregate in M20M enhances both flexural and compressive strength over time due to improved interlocking and stiffness, whereas the porous ceramic aggregate in M20B limits strength development despite the potential benefits of internal curing.

### 4.2. Shrinkage Measurement

#### 4.2.1. Standard Linear Shrinkage Test

**Figure 17:**
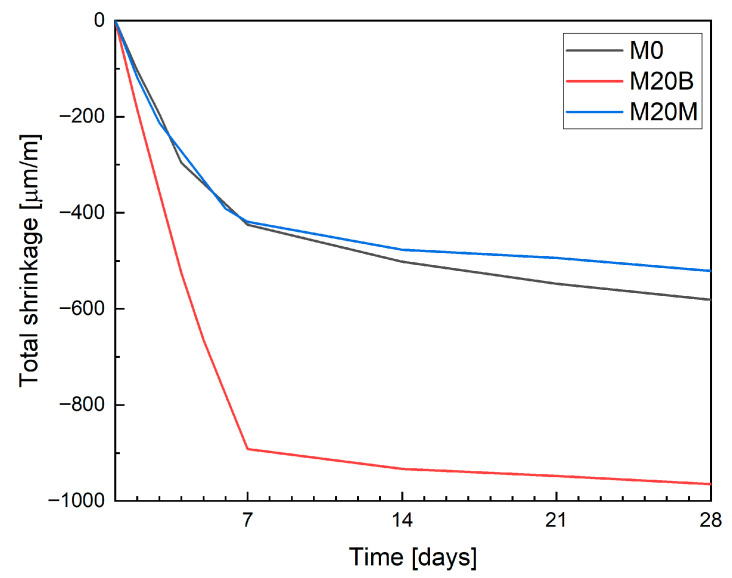
Standard linear shrinkage test results.

The total shrinkage results obtained using the linear measurement method, shown in Figure 17, represent the mean of three specimens. It should be noted that in this method, conventionally cast specimens were used, and the first measurement was performed after 24 h. Consequently, any deformations occurring during the transition from the plastic to the hardened state were not recorded. Distinct differences can be observed between the mixtures. The use of natural aggregates (sand or magnetite) did not significantly affect the measurement results. In contrast, the application of ceramic aggregate, despite the previously demonstrated internal curing effect, resulted in increased shrinkage strains. This behavior can be primarily attributed to the reduction in material stiffness, as discussed in Section 4.1.5.

#### 4.2.2. DIC with Commercial Setup

**Figure 18:**
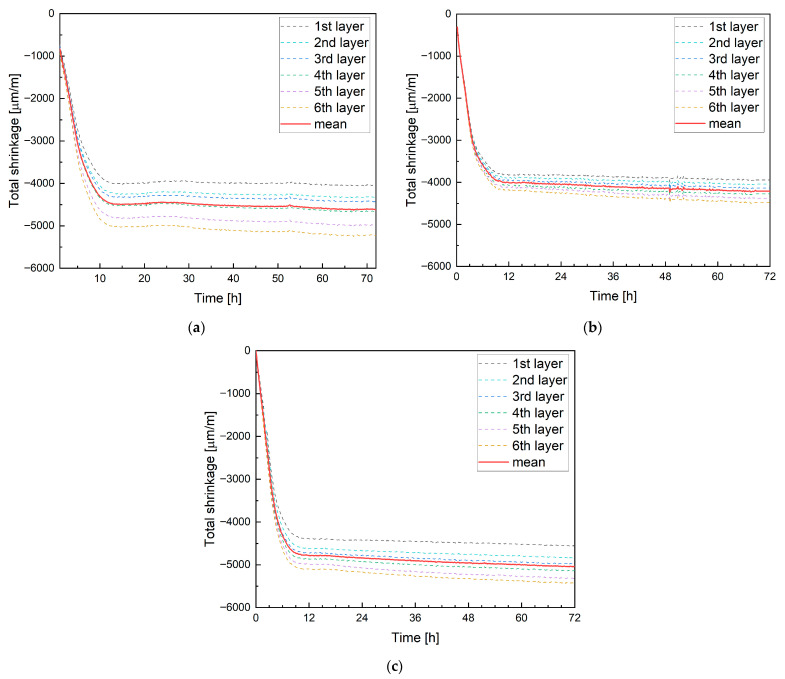
Aramis system shrinkage measurement results: (**a**) M0; (**b**) M20B; (**c**) M20M.

The results of DIC analysis using the Aramis system for mixture M0 are presented in Figure 18a (mixtures M20B and M20M are shown in Figure 18b and Figure 18c, respectively). The measurements were recorded at a frequency of one data point every 10 min. Strains were monitored for each layer, denoted as the 1st (lowest) and 6th (top) layers.

The results clearly indicate differences in strain magnitude between individual layers while maintaining similar proportional relationships. Additionally, Figure 18 shows the average strain across the entire specimen, which closely matches the strain measured in the fourth layer. Therefore, it can be concluded that the average shrinkage strain of printed elements is approximately equal to the strain measured at mid-height of the specimen. A very rapid increase in strain was observed during the first 12 h, reaching an average value of −4458 µm/m from M0. After 24 h, a distinct reduction in the strain growth rate and subsequent stabilization were recorded.

In mixture M20B containing ceramic aggregate, water absorption-desorption can be observed during the early stages of hardening. The average strain after 12 h was reduced to −4010 µm/m, corresponding to approximately a 10% decrease compared to the reference mixture. The use of magnetite aggregate (M20M) resulted in increased shrinkage, with an average strain of −4780 µm/m after 12 h. All mixtures showed a similar strain evolution pattern, with the strain growth rate slowing and stabilizing after about 12 h. Measurements extended to 72 h showed that the strain increase between 12 h and 72 h amounted to 3.6%, 5.0%, and 5.3% for the tested mixtures, respectively.

#### 4.2.3. Digital Image Correlation with Alternative Camera Systems

**Figure 19:**
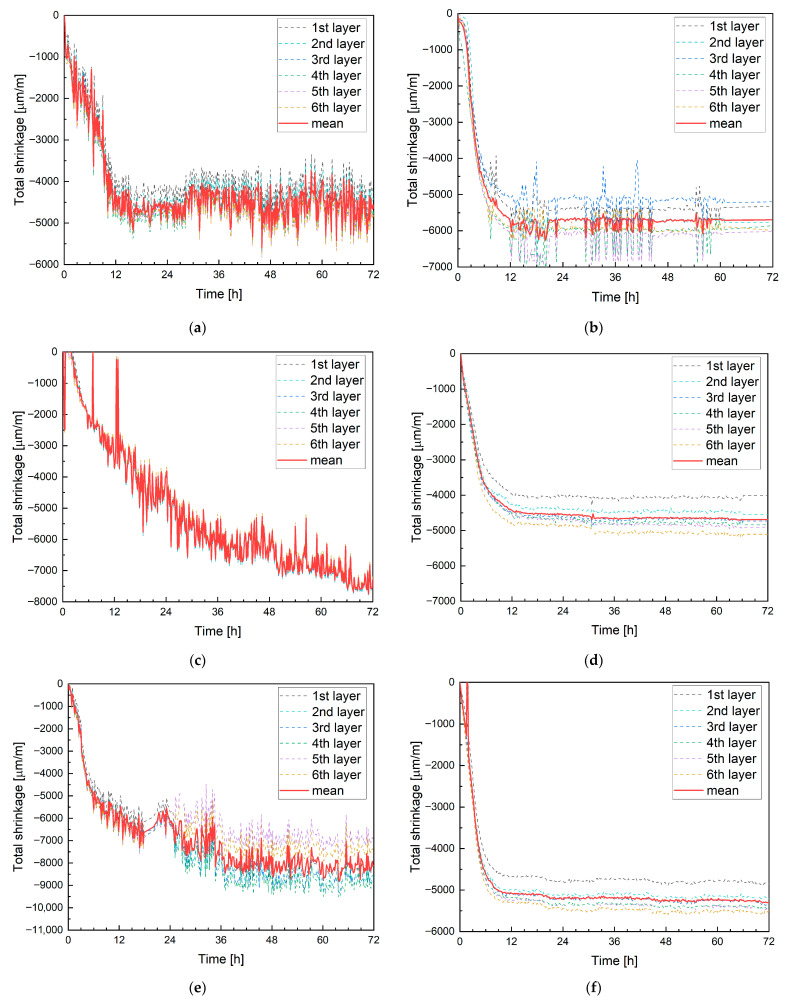
Alternative system shrinkage measurement results of mixtures: (**a**) M0 smartphone; (**b**) M0 GoPro, (**c**) M20B smartphone; (**d**) M20B GoPro, (**e**) M20M smartphone; (**f**) M20M GoPro.

For alternative image analysis solutions, a standard smartphone camera with a time-lapse application (capturing images at 10 min intervals) and a GoPro sports camera were used. Figure 19 presents the results for mixtures M0, M20B and M20M: the left side shows measurements obtained using the smartphone, and the right side shows data recorded with the GoPro camera. In both cases, increased measurement deviations are observed, manifested as local fluctuations in strain values. These fluctuations are significantly higher for the smartphone-based measurements.

Despite this, both measurement systems exhibit a similar trend, with strain stabilization observed after approximately 12 h. A notable difference, however, is the recorded shrinkage value after 12 h: for the smartphone, it was −4700 µm/m, which is close to the value measured by the Aramis system (Figure 18), while the GoPro measurement indicated a shrinkage of 5772 µm/m. It should be emphasized that both systems monitored the same specimen; therefore, the discrepancies result from image distortion caused by the camera lenses.

For mixture M20B, the smartphone measurement once again proved highly problematic. Significant noise and light reflections were observed, adversely affecting measurement quality. It is shown in Figure 19c. In addition, compared to previous measurements, the shrinkage curve shows noticeable distortions. Similar issues were not observed for the GoPro camera (Figure 19d), which recorded a shrinkage of −4426 µm/m after 12 h.

For mixture M20M (Figure 19e,f), difficulties in strain registration with the smartphone persisted. The measurements required extensive filtering and post-processing, as some measurement points were unreadable or outside the scale. In contrast, the GoPro camera provided stable and consistent data acquisition. Only minor issues occurred when capturing points on the uppermost, sixth layer; such points had to be manually excluded from the analysis. After 12 h, the average shrinkage was −5071 µm/m, a value close to that obtained with the Aramis system, though not entirely consistent.

#### 4.2.4. Laser Sensor Measurement

**Figure 20:**
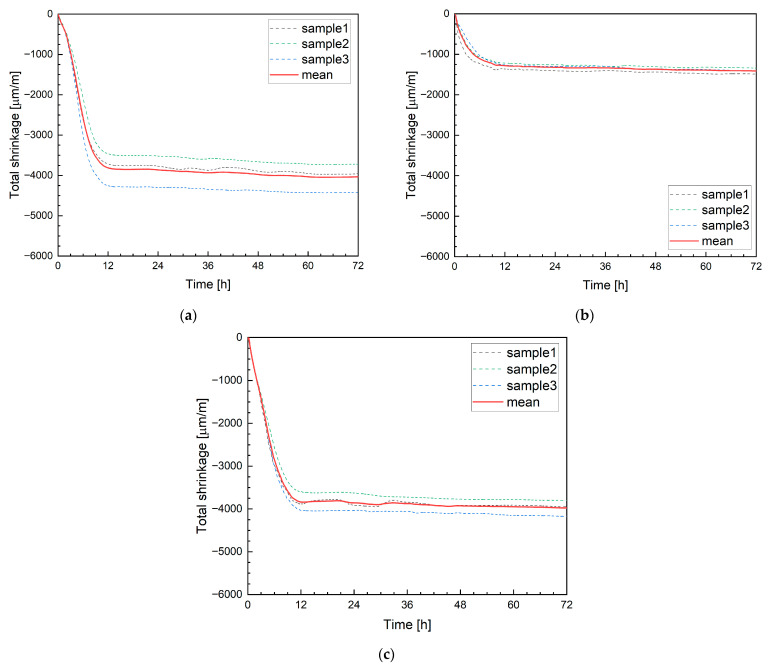
Laser system shrinkage measurement results: (**a**) M0; (**b**) M20B; (**c**) M20M.

When using laser displacement sensors, the specimens initially had to be placed in a climatic chamber due to equipment limitations. In the climatic chamber, the air relative humidity was much higher, about 70%, compared to the regular laboratory (RH = 30%), where the other tests were carried out. Additionally, the specimens were 1350 mm long, compared to 500 mm used in other measurement methods. These differences in environmental conditions and specimen geometry directly affect the evaporation rate and, consequently, the magnitude of early-age shrinkage, and therefore do not allow a direct quantitative comparison with results obtained under ambient laboratory conditions. Despite these constraints, the laser-based method demonstrated high repeatability. In contrast to the DIC techniques, measurements were performed simultaneously on three specimens. Figure 20a–c present the average shrinkage values obtained from these measurements. The figures show the shrinkage development for the mixtures M0, M20B, and M20M. The recorded strain behavior is consistent with previous measurements; the lower shrinkage values observed for the laser measurements should be interpreted primarily as a physical consequence of the higher relative humidity rather than as an effect of the measurement technique itself. For mixture M20B, noticeably lower shrinkage values were observed, which is attributed to the combined influence of reduced evaporation and internal curing effects. After 12 h, the average total shrinkage for the mentioned mixtures was −3812 µm/m, −1283 µm/m, and −3840 µm/m, respectively.

#### 4.2.5. LVDT-Based Shrinkage Measurement

**Figure 21:**
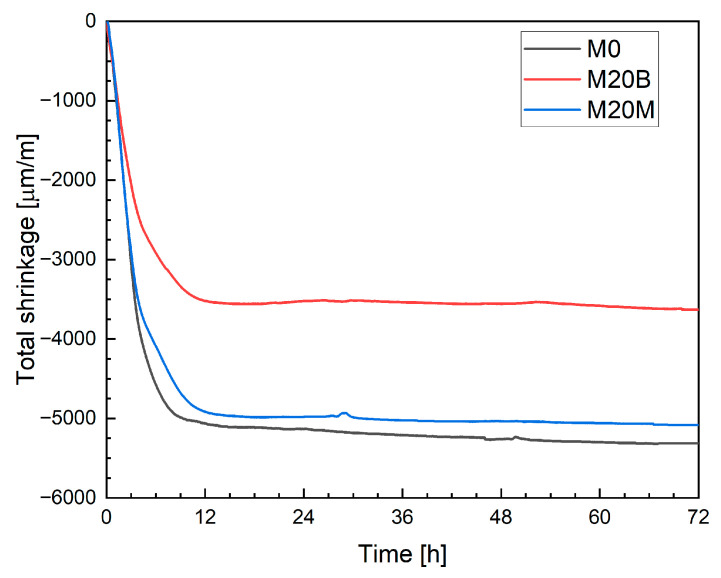
LVDT system shrinkage measurement results.

When using the LVDT sensor, the average total strain of the specimen was recorded, similarly to the laser displacement method. No measurement errors were observed, and the results were consistent, as indicated by the smooth curve shown in Figure 21. It should be noted that the LVDT setup required the insertion of a rigid bracket into the freshly printed element. Although the insert was made of plastic rather than metal to reduce mass and stiffness contrast, its presence may have locally influenced the strain field. However, no visible cracking, delamination, or surface damage was observed around the insertion point in any of the tested specimens.

Once again, mixtures M0 and M20M exhibited comparable strain values, reaching −5060 µm/m and −4916 µm/m, respectively, after 12 h. For mixture M20B, an apparent internal-curing effect was observed, resulting in a total shrinkage of −3512 µm/m. In all cases, the strain development trend was consistent with the DIC results. Nevertheless, small differences between LVDT and non-contact measurements may partly result from the invasive nature of the LVDT method, which represents a potential source of uncertainty when applied to layer-based 3D-printed elements.

## 5. Discussion

### 5.1. Material Characterization

When analyzing shrinkage results from different measurement techniques, it is important to recognize that observed differences between mixtures may arise not only from the measurement method but also from the material properties of each composition. The density of all tested materials was relatively similar, suggesting that this parameter does not significantly affect the overall deformability of the printed concrete.

However, the measured air content in the fresh mixtures, as well as the open porosity of the hardened material, clearly indicate that the use of recycled aggregate introduces additional air into the mix. This increase in entrained air reduces material stiffness, as confirmed by hardened elastic modulus tests. Across all these measurements, the M20B mixture exhibited significant deviations from the reference mixture M0, reflecting the influence of the porous recycled aggregate on the material’s mechanical response.

Calorimetric analysis further revealed a shift and delay in the hydration peak for M20B, attributed to the internal curing effect from the gradual release of water stored within the porous aggregate. This phenomenon moderates hydration kinetics, reducing self-desiccation rates and mitigating early-age shrinkage. Similar effects have been widely reported in the literature, where lightweight or recycled aggregates function as internal water reservoirs, improving volumetric stability during early hydration stages.

In the context of 3D printing, an equally important parameter is the green strength, defined as the early structural build-up of the fresh material capable of supporting subsequent printed layers. This property can indirectly influence the measurement of plastic shrinkage. When a mixture exhibits low early stiffness, multilayer printing may induce additional deformation related to creep under the weight of the upper layers. These time-dependent deformations can contribute to an apparent reduction in the measured total shrinkage, particularly when shrinkage is assessed as the net linear shortening of the specimen. This interaction among rheological stability, early-age creep, and measured shrinkage underscores the need to couple shrinkage monitoring with rheological and buildability assessments in 3D-printed concretes.

From a mechanical standpoint, compressive and flexural strength tests should be considered supplementary to shrinkage measurements, as they do not directly describe material deformability. Nevertheless, the rate of strength development in printed concrete provides valuable insight into the progression of shrinkage over time. The results indicate that the most rapid changes occur within the first few days of curing, with the three-day compressive strength reaching approximately 65% of the final value. It confirms that the early hydration stage is critical for determining both mechanical performance and volumetric stability of printed concrete.

### 5.2. Shrinkage Behavior

Regarding the deformation behavior itself, the early-age shrinkage of the three printed mixtures (M0, M20B, and M20M) was evaluated using five complementary techniques: the Digital Image Correlation (DIC) system (Aramis), DIC with consumer-grade cameras (smartphone and GoPro), laser displacement sensors, LVDT sensors, and the standardized LST method. This comprehensive approach enabled correlation of the magnitude and kinetics of shrinkage with the material composition and the accuracy of the measurement techniques used.

Each method provided distinct insights into the early-age deformation process. The DIC-based systems enabled the identification of spatial variations in strain, particularly relevant for layer-by-layer printing, while the contact sensors (LVDT, laser) ensured high repeatability and accuracy in capturing total linear deformation. The combined analysis of these datasets provides a more complete understanding of the interplay among material design, internal curing, and early-age volumetric behavior in 3D-printed concretes.

**Figure 22:**
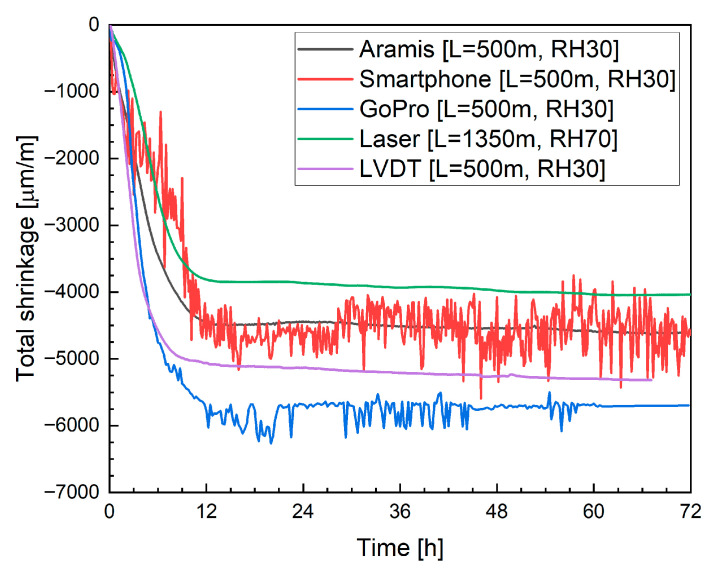
Comparison of results for different measuring methods for M0.

Figure 22 compares the shrinkage results obtained with different measurement techniques for mixture M0. The data recorded with the Aramis DIC system indicated an average shrinkage of approximately −4458 µm/m after 12 h. At that point, an apparent reduction in the rate of strain development and stabilization of the curve was observed. This time window coincides with the main hydration heat peak, and therefore, a temporary contribution of thermal expansion counteracting autogenous shrinkage cannot be fully excluded. Between 12 h and 72 h, the increase in shrinkage was limited to only 3.63%, confirming that most volumetric changes occurred during the early hydration phase. Moreover, as the printed elements were thin filamentary structures rather than massive members, the development of large internal temperature gradients is unlikely. The absence of renewed shrinkage after thermal equilibrium is reached further suggests that the observed plateau reflects a genuine stabilization of shrinkage rather than masking by thermal strains. A comparison with the literature shows that neglecting plastic deformation measurements, as in the studies by Bekaert et al. [17] and Zhang et al. [20], leads to a significant underestimation of total shrinkage. In the cited works, deformations measured on hardened specimens after one month reached only about 1000 µm/m. In contrast, the results obtained in the present study are consistent with the observations reported by Moelich et al. [21,22], where shrinkage values in the range of 6000 to 10,000 µm/m were recorded, depending on the test conditions. Moreover, the overall evolution and shape of the shrinkage curves are comparable. The shrinkage development during the first 24 h and the recorded deformation levels are also in agreement with the results reported by Federowicz et al. [33], who measured more than 4500 µm/m after 12 h, although their measurements were performed on a single-layer printed element.

The shrinkage evolution measured using the LST method exhibited a distinctly different behavior, characterized by a pronounced increase during the first seven days of curing. Moreover, the absolute shrinkage values obtained from the LST were significantly lower than those measured on 3D-printed specimens. This discrepancy results primarily from the later initiation of measurements in the LST procedure (after demolding) and the absence of early-age shrinkage registration immediately following deposition.

In the case of consumer-grade optical systems, the smartphone-based measurement yielded a value of −4700 µm/m after 12 h, whereas the GoPro camera recorded a considerably higher shrinkage of −5772 µm/m. The increased scatter and higher apparent deformation are attributed to image instability resulting from the use of automated camera settings, such as autofocus and automatic exposure, combined with optical distortions associated with wide-angle lenses. These effects reflect the limitations of using default, fully automated configurations rather than inherent limitations of the camera sensors themselves.

Measurements obtained with laser displacement sensors were initially conducted in a climatic chamber due to constraints in the instrument’s configuration, using elongated specimens. The recorded mean strain after 12 h for mixture M0 was −3812 µm/m. However, the higher relative humidity maintained in the chamber compared together with the different specimen geometry, likely contributed to the reduced shrinkage values compared to those measured under ambient laboratory conditions. Under such conditions, a direct quantitative comparison between laser-based measurements and DIC results is not justified, as the measurand itself is affected by boundary conditions and specimen length. Nevertheless, the obtained strain levels remain consistent with previously published results. In particular, Federowicz et al. [33] reported early-age shrinkage values of approximately 4500 µm/m for single-layer printed specimens, which is of the same order of magnitude as the deformations measured in the present study for multilayer elements. This agreement indicates that the deformation behavior of multilayer specimens is consistent with that observed for single-layer configurations. It is also worth emphasizing that, although the absolute shrinkage values differ due to humidity and geometry effects, the temporal evolution and overall trend of deformation development remain consistent across all measurement methods, supporting the validity of the observed shrinkage behavior.

The LVDT-based measurements, conducted under standard laboratory conditions, yielded a shrinkage value of −5060 µm/m for the same mixture. The differences between the LVDT and laser sensor results are primarily due to variations in specimen geometry and environmental conditions during testing. Similarly, discrepancies between the DIC-Aramis and DIC-GoPro (or smartphone) results arise from differences in optical configuration, image resolution, and frame stability.

Despite these variations in magnitude, the overall shape of the shrinkage curves and the time of stabilization were consistent across all measurement techniques. It should also be noted that measurements were taken on different specimens (except the smartphone and GoPro), and that variability can be significant. This convergence confirms the reproducibility of the general deformation trend, while simultaneously highlighting the sensitivity of absolute shrinkage values to measurement configuration and environmental factors.

**Figure 23:**
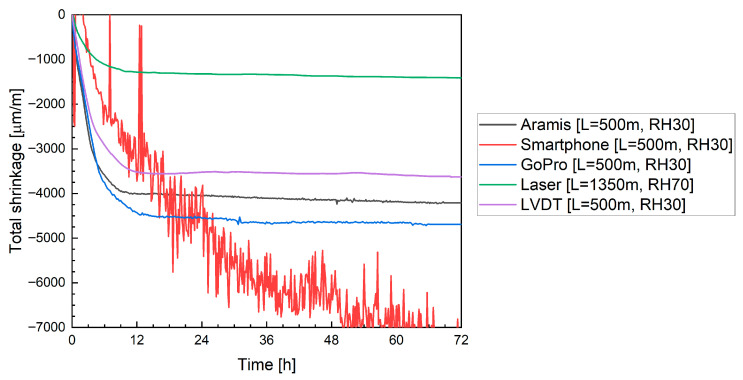
Comparison of results for different measuring methods for M20B.

A similar comparison is presented in Figure 23 for mixture M20B. Using the Aramis DIC system, an apparent reduction in shrinkage was recorded, reaching approximately −4010 µm/m after 12 h. For this mixture, the smartphone-based measurement did not yield reliable data due to excessive signal noise and light reflections, which compromised image stability and correlation accuracy. The GoPro camera produced a shrinkage value of −4426 µm/m after 12 h, showing a similar trend to the Aramis results but with larger fluctuations and reduced precision.

The laser displacement method raised the most significant concern, as it produced a significantly lower shrinkage value of −1283 µm/m. It could be caused by additional friction between the sample and the printing platform, as laser sensors used longer samples (1350 mm). As discussed earlier, both the higher relative humidity and the modified specimen geometry influence the evaporation rate and lead to a different development of internal stresses within the specimen, which directly affects the measured shrinkage magnitude. Despite the reduced magnitude, the shape of the deformation curve and its temporal evolution remain consistent with those obtained using the other measurement methods, indicating comparable shrinkage kinetics. This suggests that the observed discrepancy is primarily a physical consequence of the specific boundary conditions in the climatic chamber, particularly the elevated relative humidity (RH = 70%), which limited water loss and thus reduced shrinkage, rather than a limitation of the laser measurement technique itself.

In contrast, the LVDT measurements demonstrated high precision and consistency with DIC methods, yielding a shrinkage value of −3512 µm/m after 12 h. The agreement between the LVDT and Aramis results supports the conclusion that mixture M20B exhibited a pronounced reduction in early-age shrinkage compared to the reference mixture, primarily due to the internal curing effect provided by the porous recycled aggregate.

For the Aramis DIC system, the shrinkage of mixture M20M increased to approximately −4780 µm/m after 12 h. Using the GoPro camera, the filtered data indicated a shrinkage of −5071 µm/m, while the smartphone-based measurement produced an even higher value of −6292 µm/m. The overestimation observed in the smartphone results is most likely due to optical distortion and light reflection effects, which introduce instability into image-based strain tracking.

**Figure 24:**
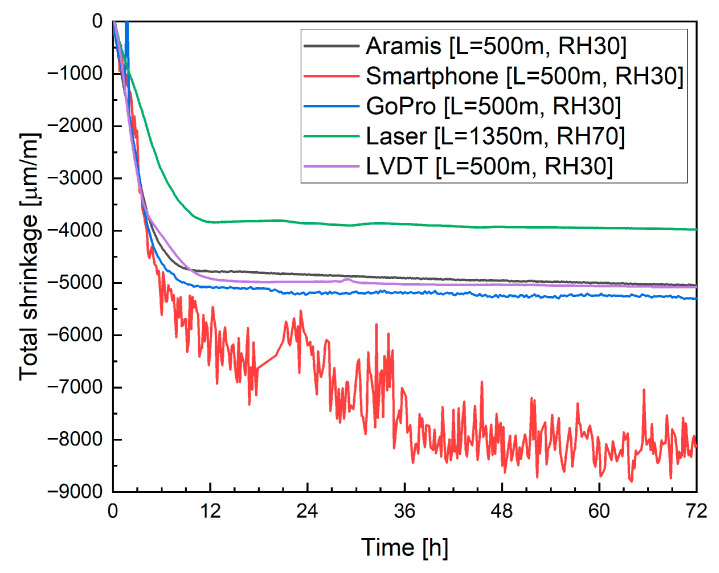
Comparison of results for different measuring methods for M20M.

Measurements obtained with laser displacement sensors showed a total deformation of −3840 µm/m, once again emphasizing the strong sensitivity of printed elements to curing conditions. In this case, the specimens were stored in a climatic chamber, where the higher relative humidity reduced water evaporation and, consequently, limited shrinkage. Summarized mean values of measured deformation are presented in Figure 24. The lower deformation measured with the laser sensor (in comparison to M0) does not contradict the observed behavior of M20M, as both the higher relative humidity and the different specimen geometry are known to modify the stress development during early-age shrinkage, while the overall deformation trend remains consistent with the other measurement methods.

The LVDT measurements yielded a total shrinkage of −4916 µm/m, which is consistent with the results from the Aramis system and the GoPro-based DIC method. For mixture M20M, the consistency across these three methods indicates a good agreement between the optical and contact measurement techniques and the material’s stable deformation behavior under controlled laboratory conditions.

The LST is not suitable for mixtures intended for 3D printing applications. In this method, measurements begin 24 h after casting on conventionally molded specimens, so neither plastic shrinkage nor a portion of autogenous shrinkage occurring during the early hydration phase is captured. This limitation is not critical for conventional concretes, for which EN 12390-16 was developed, but it becomes significant in the case of 3D-printed elements. In such elements, the first 24 h are characterized by rapid moisture exchange with the environment and the absence of formwork confinement, both of which strongly affect the development of early-age deformations. Even though Table 2 compares different measuring methods, the presented values represent only the incremental total deformation between 24 h and 72 h. For mixture M20B, higher total long-term deformations were recorded, which the authors attribute to the material’s reduced stiffness despite the presence of internal curing effects.

However, when compared with optical measurements (Aramis), the LST results capture only a limited fraction of the total shrinkage observed at 72 h, indicating that a substantial part of the deformation develops before the start of the LST measurement. To quantify this effect, the percentage difference between the total shrinkage measured at 72 h using Aramis and LST was calculated (Table 3). This “missing fraction” explicitly represents the portion of deformation not captured by EN 12390-16 when applied to 3D-printed mixtures. The results show that, while the LST method may still be indicative of deformation trends in the hardened phase, it does not adequately represent the overall early-age shrinkage behavior of layer-by-layer printed materials.

### 5.3. Methods Comparison

To enable a consistent comparison of different shrinkage measurement techniques, all methods were applied under identical boundary conditions and on the same printed specimens. The objective of this comparison stage was not to establish statistical equivalence between methods, but to verify their consistency in capturing early-age deformation trends under controlled conditions and to assess their practical comparability when applied to the same printed element. For this reason, an attempt was made to perform all types of measurements on the same specimen under constant temperature and humidity conditions. To achieve this, the experimental setup for shrinkage measurements using laser displacement sensors was modified, and the specimen length was reduced from 1350 mm to 500 mm. This reduction allowed the application of multiple measurement techniques within a single experimental configuration while maintaining comparable boundary conditions.

Due to technical limitations, it was not possible to simultaneously measure both laser sensors and LVDT transducers on a single specimen. Therefore, an additional specimen was prepared specifically for the LVDT measurements. This supplementary specimen was not intended for statistical comparison, but solely for a qualitative cross-check of measurement consistency. This supplementary specimen was placed in the same laboratory environment, directly adjacent to the primary sample, ensuring identical thermal and hygric conditions.

**Figure 25:**
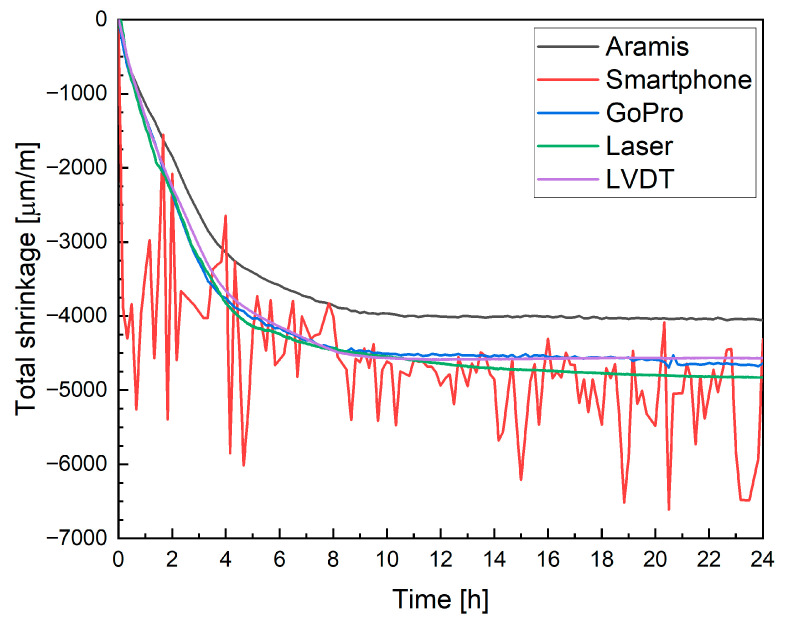
Comparison of results obtained using different measurement methods for M20B on the same specimen.

Mixture M20B was selected for this stage of testing because it had previously shown the most significant variability in measurement results and thus provided the best basis for evaluating the consistency and comparability of the measurement methods. The corresponding results are presented in Figure 25.

Furthermore, the measurement duration was limited to 24 h. This decision was based on earlier findings showing that after the first 12 h, the deformation rate significantly decreases and the shrinkage curve stabilizes. Extending the measurement beyond 24 h, therefore, provided limited additional insight into early-age deformation behavior while unnecessarily prolonging the test.

When variable conditioning conditions and differences in specimen geometry (length) were eliminated, the obtained results became highly consistent in terms of deformation trends and magnitudes, supporting good repeatability within the scope of the present comparison. This agreement supports the internal consistency of the applied measurement methods within the scope of the presented study. The smartphone camera in automatic setting, however, showed limited suitability for such measurements due to its built-in autofocus, which continuously adjusts the focal depth and introduces slight image shifts. This limitation reflects the use of fully automated camera settings rather than an inherent constraint of smartphone sensors. In high-precision strain monitoring, even small displacements can significantly affect the accuracy of correlation analysis, leading to substantial measurement errors.

In contrast, the DIC results obtained with both the Aramis system and the GoPro camera showed very close agreement. In fact, the measurements performed using the DIC method with the GoPro camera, as well as those from the laser displacement sensors and the LVDT transducers, exhibited nearly identical strain evolution curves. The Aramis system produced slightly different absolute values, which can be attributed to its averaging strain across several measurement layers rather than tracking a single linear segment.

Additionally, for DIC measurements, the definition of the initial reference scale is a critical factor. Since the registered strain values are relatively small, even minor inaccuracies in setting the initial scale can result in deviations of up to 10% in the final computed shrinkage. This observation highlights the importance of precise calibration procedures and fixed camera positioning when using DIC for early-age shrinkage monitoring in 3D-printed concrete.

In summary, sensor-based methods, such as laser displacement transducers or LVDT gauges, measure the average strain along the specimen’s length and provide high measurement repeatability. These techniques are reliable for determining total linear deformation but do not capture local variations along the printed layers.

The DIC method implemented in the Aramis system, on the other hand, allows spatially resolved analysis of deformation across individual printed layers. This capability makes it particularly valuable for investigating interlayer behavior and localized strain concentration zones. However, DIC requires strict control of lighting conditions, precise calibration, and stable geometry throughout the test. Even minor variations in illumination, camera angle, or reference scale definition can introduce measurable discrepancies in the results.

Consumer-grade cameras, such as smartphones or GoPro devices, may serve as valuable tools for preliminary or comparative testing, offering a low-cost approach to visual strain monitoring. Nevertheless, their measurement accuracy is significantly limited by optical distortions and automated camera functions, including autofocus and exposure correction. These issues can be mitigated mainly by turning off automatic adjustments and setting manual focus and exposure parameters before testing.

These observations align well with the literature on the use of DIC techniques for monitoring early-age deformation in cementitious materials. Studies consistently emphasize that reliable quantitative results from DIC require rigorous experimental control, stable environmental conditions, and consistent calibration protocols. Under these conditions, DIC provides a practical and non-invasive alternative for assessing early shrinkage behavior in 3D-printed concrete systems [34,35].

When comparing the results obtained for the tested mixtures, it is important to emphasize that, with appropriately selected measurement techniques, it is possible to capture and quantify the mechanism of internal curing in 3D-printed concrete elements. The porous, presaturated aggregate acts as an internal water reservoir, gradually releasing moisture during hydration. This mechanism mitigates self-desiccation and reduces autogenous shrinkage, thereby improving the volumetric stability of the printed material during the critical early stages of curing.

The effectiveness of water transfer using lightweight or recycled aggregates has already been demonstrated in numerous studies on conventionally cast concrete [36,37,38]. These works confirm that the gradual release of water from porous inclusions promotes a more uniform hydration front, delays the formation of capillary stress, and limits microcrack development. However, comprehensive studies addressing this phenomenon specifically in 3D-printed concrete elements remain scarce.

This knowledge gap is significant because the layer-by-layer deposition process introduces unique material and structural conditions that may alter internal curing dynamics—particularly the anisotropic pore structure, directional build-up, and differential surface exposure to evaporation. Therefore, adopting advanced measurement methods, such as DIC, laser displacement sensors, or LVDT transducers, for printed concrete offers a valuable opportunity to monitor and better understand how internal curing affects shrinkage behavior in additively manufactured cementitious systems.

The results of this study confirm that the total shrinkage observed during the first hours of curing in 3D-printed concrete elements is substantial. Similar observations have been reported in previous research. Moelich et al. [22] reported plastic shrinkage values of approximately 6000 µm/m in 3D-printed concrete elements, noting that the risk of cracking was significantly higher than in conventionally cast concrete. Likewise, Markin and Mechtcherine [35] presented a comparable experimental setup for plastic shrinkage measurement to that used in the present study, reporting deformation levels approaching −9000 µm/m.

These findings support the conclusion that the first 12 h after deposition constitute a critical window for deformation development in printed concrete. During this period, both plastic and autogenous shrinkage evolve rapidly, directly influencing the structural integrity and interlayer bond quality of printed elements. Continuous monitoring under controlled environmental conditions is therefore essential for accurately capturing this phase.

The importance of such early-stage monitoring was also highlighted by Mechtcherine et al. [24], who emphasized the need to adapt quality control methods to the specific requirements of 3D-printed materials. The authors pointed out that real-time strain tracking and layer-by-layer deformation recording should become integral parts of standard testing protocols for additive manufacturing with cementitious materials.

The measurement strategy presented in this paper aligns with these recommendations by combining both averaged and surface-based monitoring techniques. The laser and LVDT sensors provide reliable, averaged strain data along the specimen’s length, while the DIC method delivers high-resolution surface deformation mapping. Integrating these complementary approaches ensures a comprehensive understanding of shrinkage mechanisms in printed concrete, enhances the credibility of the experimental findings, and supports the broader development of standardized testing methodologies for 3D-printed cementitious systems. A synthetic summary of the study findings is presented in Table 4. In Table 5, a relative scale from 1 to 5 (1 = lowest) is used to provide an indicative assessment of the cost, accuracy, and ease of application of each measurement method.

Measurement precision, understood as the resolution of the recorded signal or image, is comparable for all DIC-based methods considered in this study. The commercial ARAMIS system records images with a resolution of 4096 × 3068 pixels, while the smartphone camera used in this study provides a resolution of approximately 5000 × 3800 pixels, and the GoPro camera records images at 4000 × 3000 pixels. From the perspective of image resolution alone, no significant differences in potential spatial precision can therefore be expected between the DIC approaches. For non-optical methods, the measurement precision is governed by the characteristics of the applied sensors. The LST method, based on a mechanical dial gauge, provides a measurement resolution of 0.001 mm. The laser displacement sensor used in this study offers a nominal accuracy of approximately 2 µm. For LVDT sensors, the nominal accuracy is not explicitly defined by the manufacturer; however, based on the observed signal stability and noise level, a conservative measurement precision of approximately 1 µm can be safely assumed.

Repeatability of the measurements is difficult to quantify explicitly due to the inherent geometric variability of 3D-printed elements and was therefore not the primary focus of the present study. Nevertheless, by comparing the shapes of the recorded deformation curves and the similarity of the measured values, a high level of repeatability can be assumed for both commercial and low-cost DIC methods (in particular, the GoPro-based setup), as well as for laser sensors and LVDT measurements. The most significant non-budget-related limitation is associated with laser sensors and LVDTs. These techniques do not allow full-field surface measurements and are limited to recording average deformations over the gauge length, which prevents the analysis of local shrinkage gradients or spatially heterogeneous deformation patterns.

Summarizing the data presented in Table 4 and Table 5, special attention should be paid to cost-related aspects. The measurement setup used for LST is inexpensive and widely available. Almost every civil engineering laboratory has such equipment. Therefore, it is a suitable tool for preliminary evaluation of material parameters for printing. However, it is not applicable to testing printed elements due to the previously discussed limitations.

Commercial DIC measurement systems are complete solutions with high measurement accuracy and good repeatability. In the literature, they are commonly regarded as reliable measurement tools. Nevertheless, their operation is complex, associated with high costs, and requires time-consuming calibration and specimen preparation.

The use of low-cost cameras, such as smartphones or GoPro devices, represents a viable alternative for preliminary and comparative studies. However, fully automatic focus and exposure settings should be avoided, as they may affect measurement quality. The use of manual control of operating parameters is recommended.

In the case of laser sensors and LVDTs, the cost of the sensors themselves is relatively low. However, it should be noted that the price indicated in Table 5 (approximate) refers to two sensors (a setup for a single test specimen) and does not include the cost of the data acquisition unit. Such an amplifier typically costs between USD 5000 and 20,000, depending on the configuration. Nevertheless, most research institutions already possess such equipment, as its versatility allows the use of various types of sensors. A limitation of laser sensors and LVDTs is that they provide only average deformation measurements of structural elements, without the possibility of analyzing local deformations.

## 6. Conclusions and Limitations

This study provides a systematic evaluation of contact and non-contact techniques for monitoring early-age shrinkage in 3D-printed concrete and leads to several practical and methodological conclusions.

The results confirm that early-age shrinkage in additively manufactured cementitious materials develops rapidly and must be monitored immediately after printing, particularly within the first 12–24 h. This behavior is governed by moisture loss, early hydration, and the absence of confinement typical of layer-by-layer fabrication. Measurement intervals of 10–15 min were shown to be sufficient to capture the dominant deformation mechanisms without excessive data redundancy.

From a methodological perspective, digital image correlation requires carefully controlled optical conditions. Stable diffuse lighting and fixed camera settings are essential to ensure reliable strain evaluation. The use of discrete surface markers instead of full speckle patterns proved effective and better suited to printed elements. Importantly, standard cameras and smartphones demonstrated sufficient accuracy when properly calibrated, indicating that low-cost optical approaches can represent viable alternatives to commercial DIC systems for early-age shrinkage assessment. The reduced performance observed for smartphone-based measurements in this study resulted primarily from the use of fully automated camera settings and should not be interpreted as an inherent limitation of smartphone imaging sensors.

Laser displacement sensors and LVDT transducers provided accurate and repeatable measurements of total linear deformation. However, these techniques do not capture localized strain fields and should be interpreted as complementary rather than equivalent to optical methods. Traditional shrinkage tests developed for cast concrete were found to be inadequate for 3D-printed materials, as they do not account for plastic and autogenous shrinkage or for the specific curing conditions associated with additive manufacturing.

The main limitations of this study are its laboratory scale, the absence of reinforcement, and the use of controlled environmental conditions. These factors may limit direct transfer of the results to real-scale printed structures exposed to variable temperature and humidity. In addition, part of the experimental program involved different environmental conditions and specimen geometries, which directly affect evaporation rate, stress development, and absolute shrinkage magnitude, thereby restricting direct quantitative comparison between measurement techniques. Nevertheless, the findings have practical relevance for laboratory testing and suggest the need for adapting existing standards or developing new guidelines dedicated to 3D-printed concrete.

Future research should focus on large-scale printed elements, variable environmental conditions, and reinforced systems. Further integration of experimental measurements with numerical models is also recommended to improve prediction of early-age deformation and to support the development of normative frameworks for additive manufacturing in concrete construction. Future studies should also include larger specimen populations to enable statistical assessment of measurement variability and method sensitivity.

## Figures and Tables

**Table 1:** Mix designs of the 3DPC tested in the study (note: quantities in kg/m^3^).

	CEM I 42.5R	Fly Ash	Silica Fume	River Sand	Magnetite Aggregate	Recycled Masonry Aggregate	SP	Water	Extra Water
M0	580	166	83	1300	–	–	2.3	200	–
M20B				1040	–	258.0	2.5		20
M20M				1040	502.2	–	2.4		–

**Table 2:** Comparison of incremental total deformation between 24 h and 72 h—LST vs. other methods (note: quantities in µm/m).

Mix	LST	Aramis	Smartphone	GoPro	Lasers	LVDT
M0	−193.75	−161.92	−99.9	76.6	−171.36	−182.0
M20B	−356.25	−152.99	−3347.8	−142.1	−89.5	−105.8
M20M	−212.5	−254.72	−1809.8	−223.1	−135.06	−166.0

**Table 3:** Comparison of total shrinkage at 72 h measured by Aramis and LST, including the estimated missing deformation fraction for LST (note: quantities in µm/m and %).

Mix	LST	Aramis	Missing Deformation
M0	−193.75	−4620.1	95.81%
M20B	−356.25	−4209.7	91.54%
M20M	−212.5	−5034.3	95.78%

**Table 4:** Comparison of measuring methods.

Method	Advantages	Disadvantages	Remarks
LST	Inexpensive and straightforward; Widely used	Does not record plastic shrinkage; Does not reflect printing conditions	Not suitable for 3DPC, omits critical early hours
Commercial DIC	Very high accuracy and resolution; Ability to analyze local deformations; Measurement from the plastic state without contact	Very high cost; Requires stable lighting and calibration; Complex operation	Most accurate method, complete spatial analysis; ideal for scientific research
DIC with a smartphone	Low cost; Easy availability	Significant measurement errors; Susceptible to lighting; Requires extensive data processing	Can be used for preliminary tests, but has low reliability
DIC with GoPro	Low cost; More stable than a smartphone; Can capture the shrinkage trend	Optical distortions; Lower precision than the commercial system	Better than a smartphone, but requires data filtering
Laser sensors	Very high accuracy (approx. 2 µm); Non-contact; Stable results	Measures only average shrinkage; Sensitive to environmental conditions; Requires special setup	Excellent repeatability, good for linear measurements
LVDT	High accuracy; Stable results; Simple interpretation	Requires contact with specimen; Does not show local deformations	Very reliable method for total shrinkage

**Table 5:** Measuring methods evaluation.

Method	Cost	Accuracy	Ease of Implementation
LST	500$	2	5
Commercial DIC	>25,000$	5	2
DIC with a smartphone	300$	2	4
DIC with GoPro	300$	3	4
Laser sensors	500$ *	5	5
LVDT	600$ *	5	3

Where 1 means the lowest value, and 5 is the highest, * price of the sensor only.

## Data Availability

The original contributions presented in the study are included in the article, further inquiries can be directed to the corresponding author.
